# Immunohistochemical detection of membrane-type-1-matrix metalloproteinase in colorectal carcinoma

**DOI:** 10.1054/bjoc.2000.1195

**Published:** 2000-06-15

**Authors:** R Kikuchi, T Noguchi, S Takeno, N Kubo, Y Uchida

**Affiliations:** Department of Surgery II, Oita Medical University, 1–1 Idaigaoka, Hasama-machi, Oita, 879–5593, Japan

**Keywords:** membrane-type-1-matrix metalloproteinase, matrix metalloproteinase-2, tissue inhibitor of metalloproteinase-2, immunohistochemistry, distant metastases, colorectal carcinoma

## Abstract

We investigated whether the expression of membrane-type-1 matrix metalloproteinase (MT1-MMP), matrix metalloproteinase-2 (MMP-2) and tissue inhibitor of metalloproteinase-2 (TIMP-2) was consistent with the proposed roles of these proteins in promoting metastasis in colorectal cancer. The expression of MT1-MMP was significantly more frequent in deeply invasive carcinomas (*P* = 0.007) and in cases of vascular invasion (*P* = 0.02). The frequency of detection of MMP-2 in the stroma was much greater in vascular invasion-positive cases (42%) than in negative cases (20%;*P* = 0.02). The rate of detection of TIMP-2 in tumour cell cytoplasm increased with the depth of invasion (*P* = 0.03). TIMP-2 in the stroma was found more frequently in tumours with lymphatic invasion and lymph node metastasis (*P*< 0.05). Significant correlations were found between detection of MT1-MMP and MMP-2 in tumour cell cytoplasm (*P*< 0.05), of MT1-MMP and TIMP-2 in tumour cell cytoplasm (*P*< 0.01), and of MMP-2 and TIMP-2 in tumour cell cytoplasm (*P*< 0.01). Immunohistochemical detection of MT1-MMP and TIMP-2 might be useful for monitoring infiltration in colorectal carcinoma but is not correlated with distant metastases. © 2000 Cancer Research Campaign


British Journal of Cancer (2000) 83(2), 215–218
© 2000 Cancer Research Campaign
doi: 10.1054/ bjoc.2000.1195, available online at http://www.idealibrary.com on IDEAL®

### Immunohistochemical detection of membrane-type-1-matrix metalloproteinase in colorectal carcinoma

**R Kikuchi, T Noguchi, S Takeno, N Kubo and Y Uchida**

Department of Surgery II, Oita Medical University, 1–1 Idaigaoka, Hasama-machi, Oita 879–5593, Japan

**Summary** We investigated whether the expression of membrane-type-1 matrix metalloproteinase (MT1-MMP), matrix metalloproteinase-2 (MMP-2) and tissue inhibitor of metalloproteinase-2 (TIMP-2) was consistent with the proposed roles of these proteins in promoting metastasis in colorectal cancer. The expression of MT1-MMP was significantly more frequent in deeply invasive carcinomas ($P = 0.007$) and in cases of vascular invasion ($P = 0.02$). The frequency of detection of MMP-2 in the stroma was much greater in vascular invasion-positive cases (42%) than in negative cases (20%; $P = 0.02$). The rate of detection of TIMP-2 in tumour cell cytoplasm increased with the depth of invasion ($P = 0.03$). TIMP-2 in the stroma was found more frequently in tumours with lymphatic invasion and lymph node metastasis ($P < 0.05$). Significant correlations were found between detection of MT1-MMP and MMP-2 in tumour cell cytoplasm ($P < 0.05$), of MT1-MMP and TIMP-2 in tumour cell cytoplasm ($P < 0.01$), and of MMP-2 and TIMP-2 in tumour cell cytoplasm ($P < 0.01$). Immunohistochemical detection of MT1-MMP and TIMP-2 might be useful for monitoring infiltration in colorectal carcinoma but is not correlated with distant metastases. © 2000 Cancer Research Campaign

**Keywords**: membrane-type-1-matrix metalloproteinase; matrix metalloproteinase-2; tissue inhibitor of metalloproteinase-2; immunohistochemistry; distant metastases; colorectal carcinoma

Distant metastasis is one of the most important determining factors in the prognosis of colorectal cancer. Degradation of the extracellular matrix that surrounds tumour cells is an essential step in the processes of tumour invasion and metastasis. Matrix metalloproteinases (MMPs) degrade various components of the extracellular matrix. In particular, MMP-2 (gelatinase A; also called type-IV collagenase and 72-kDa gelatinase) degrades type IV collagen of the basement membrane, as well as gelatin, and type V, VII and X collagens (Collier et al, 1997). MMP-2 is expressed in different types of human epithelial cancer, such as breast (Davies et al, 1993), ovarian (Naylor et al, 1994), pancreatic (Bramhall et al, 1996), and gastric (Sier et al, 1996) cancer, and its level seems to be related to malignancy and invasion. The activity of MMP-2 is modulated by a tissue inhibitor of metalloproteinase-2 (TIMP-2) (Hayakawa, 1994; Stetler-Stevenson et al, 1993*a*). Moreover, there have been several reports that the level of MMP-2 is not related to malignancy and invasion (Visscher et al, 1994; Ring et al, 1997) and that it is the level of TIMP-2 that is related to these processes (Visscher et al, 1994; Ring et al, 1997; Murashige et al, 1996). These results contradict the original proposed functions of MMP-2 and TIMP-2. In addition, membrane-type-1 matrix metalloproteinase (MT1-MMP) was recently identified (Sato et al, 1994). Conflicts among the above results might be resolved by studies of the functional interplay between MT1-MMP, MMP-2 and TIMP-2. The primary aim of the present study was to investigate whether the results of immunohistochemical detection of MT1-MMP, MMP-2 and TIMP-2 in colorectal cancers might be consistent with the proposed roles of these proteins in the promotion of the metastatic behavior of tumours.

*Received 9 September 1999*
*Revised 18 February 2000*
*Accepted 24 February 2000*

*Correspondence to:* R Kikuchi

### MATERIALS AND METHODS

### Patients and tumour samples

A total of 92 adenocarcinomas of the colon and rectum were studied. Tumours were obtained surgically between 1988 and 1993 at the Department of Surgery II, Oita Medical University. There were 11 cases of simultaneous distant metastasis and nine cases of allochronic distant metastasis. All specimens were fixed in 10% buffered formalin and embedded in paraffin. The tumours were staged according to the standard TNM classification (Sobin and Wittekind, 1997).

### Immunohistochemistry

For immunohistochemical analysis, 4 µm-thick sections were cut from formalin-fixed, paraffin-embedded blocks and placed on silan-coated slides. After deparaffinization, the sections were incubated in 3% hydrogen peroxide for 20 min in order for devitalization of the peroxidase. Deparaffinized and rehydrated specimens were heated in 10 mM citrate buffer, pH 6.0, for 10 min in an autoclave at 121°C. After cooling to room temperature (RT) for 30 min, the specimens were incubated with normal rabbit serum for 15 min at RT. Then they were incubated with various primary antibodies, namely, monoclonal antibody against MT1-MMP (114–6G6, 1:25; Fuji Chemical Industries, Japan), monoclonal antibody against MMP-2 (42-5D11, 1:100; Fuji Chemical Industries) or monoclonal antibody against TIMP-2 (67-4H11, 1:100; Fuji Chemical Industries) for 16 h at 4°C. After incubation, immunohistochemical staining was performed by the standard avidin-biotin-peroxidase complex (ABC) technique with an LSAB kit (Nichirei, Tokyo, Japan) and 3,3′-diaminobenzidine as the chromogen. Nuclei were counterstained with haematoxylin. For evaluation of immunohistochemical staining, specimens were divided into two groups as follows. The immunopositive cell area was used for the evaluation of the immunohistochemical staining of MT1-MMP, MMP-2 and TIMP-2 monoclonal antibody: negative, 0–10%; positive, > 10%. Expression of MMP-2 and TIMP-2 was also evaluated in terms of immunostaining of the tumour cell cytoplasm and the stroma. A clinicopathologic study was performed by reference to the depth of invasion, lymphatic invasion, venous invasion, lymph node metastasis, and distant metastasis.

### Statistical analysis

Correlations between the expression of each antigen and the various clinicopathologic factors were examined by the χ-squared test, Fisher's exact probability test and Mann-Whitney's U-test. Furthermore, correlations between the expression of pairs of antigens were studied by the χ-squared test.

### RESULTS

### Immunohistochemical staining of MT1-MMP, MMP-2 and TIMP-2

Immunohistochemical staining indicated that MT1-MMP was localized predominantly in the tumour cell cytoplasm and it was weakly or not expressed in normal tissue (Figure 1A). The frequency of samples positive for MT1-MMP was 36% (33/92). MMP-2 (Figure 1B) and TIMP-2 (Figure 1C) were localized by immunostaining in the tumour cell cytoplasm and the stroma. The frequency of samples positive for MMP-2 in the tumour cell cytoplasm was 20% (18/92) and in the stroma it was 30% (27/92). Thirteen samples (14%) in 92 were positive for MMP-2 in both the tumour cell cytoplasm and the stroma. The frequency for samples positive for TIMP-2 in the tumour cell cytoplasm was 32% (29/92) and in the stroma it was 47% (43/92). Eighteen samples (20%) in 92 were positive for TIMP-2 in both the tumour cell cytoplasm and the stroma.

### Correlations between the expression of each antigen and the various clinicopathologic factors

Table 1 shows the correlations between the expression of each antigen and the various clinicopathologic factors. The frequency of immunodetection of MT1-MMP increased with increases in the depth of invasion ($P$ = 0.007; Mann-Whitney U-Test). The percentage of MT1-MMP-positive cases was significantly higher in vascular invasion-positive cases (54%) than in invasion-negative cases (29%; $P$ = 0.02; χ-squared test). The percentage of cases positive for MMP-2 in the stroma was significantly higher in vascular invasion-positive cases (42%) than in invasion-negative cases (20%; $P$ = 0.02; χ-squared test). The frequency of detection of TIMP-2 in the tumour cell cytoplasm increased with increases in the depth of invasion ($P$ = 0.03; Mann-Whitney U-test). TIMP-2 was detected in the stroma more frequently in tumours with lymphatic invasion and lymph node metastasis ($P$ < 0.05; χ-squared test) than in tumours without such features.

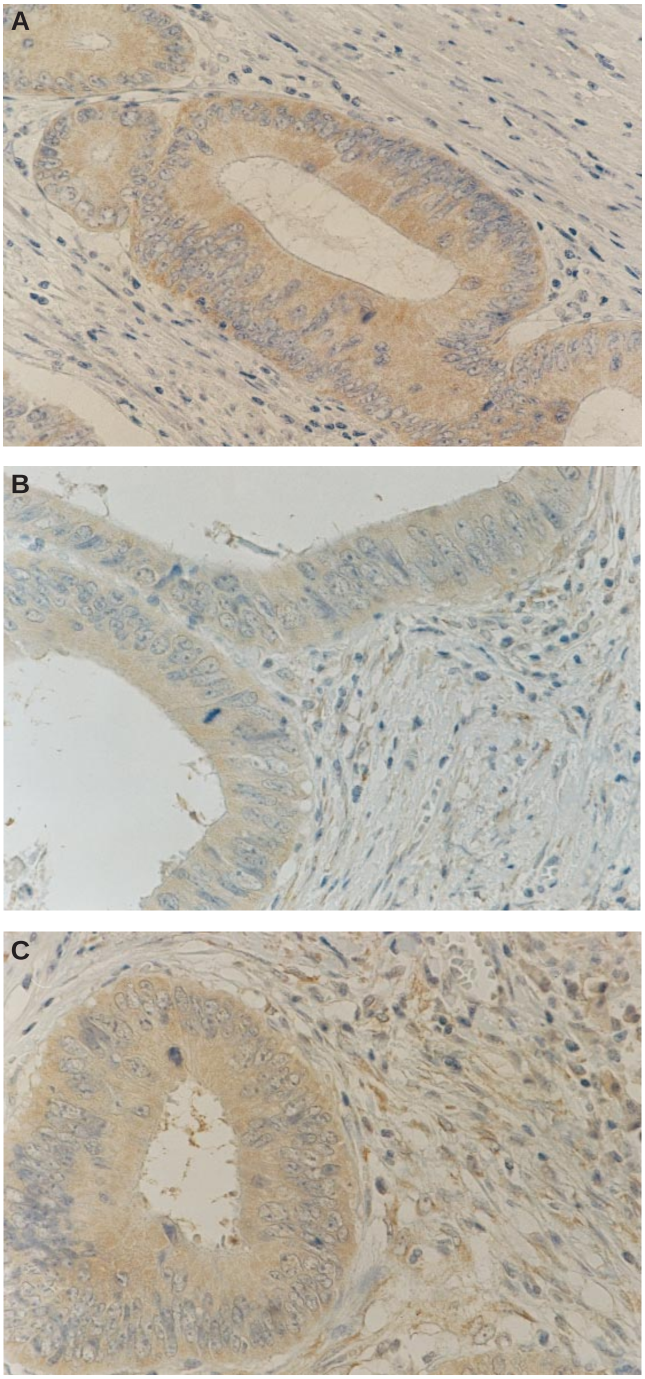

**Figure 1** Immunolocalization of MT1-MMP (**A**), MMP-2 (**B**) and TIMP-2 (**C**) in samples of human colorectal carcinomas. Immunostaining was performed with monoclonal antibodies raised against MT1-MMP, MMP-2 and TIMP-2 as described in Materials and methods. Immunostaining revealed MT1-MMP is in the cytoplasm of tumour cells (**A**), while MMP-2 (**B**) and TIMP-2 (**C**) were immunostained in the tumour cell cytoplasm and the stroma, respectively (original magnification × 400).

### Correlations among antigens

The percentage of cases positive for MT1-MMP was significantly higher in cases positive (61%, 11/18) for MMP-2 in the tumour cell cytoplasm than in negative cases (30%, 22/74; $P$ = 0.013; χ-squared test). The percentage of cases positive for MT1-MMP was

 © 2000 Cancer Research Campaign

**Table 1** Correlations between the expression of MT1-MMP, MMP-2 and TIMP-2 and clinicopathologic factors

| | MT1-MMP | | MMP-2 | | | | TIMP-2 | | | |
|---|---|---|---|---|---|---|---|---|---|---|
| | | | Tumour cell cytoplasm | | Stroma | | Tumour cell cytoplasm | | Stroma | |
| Variable | Positive rate (%) | *P* value | Positive rate (%) | *P* value | Positive rate (%) | *P* value | Positive rate (%) | *P* value | Positive rate (%) | *P* value |
| Depth of invasion[a] | | | | | | | | | | |
| T1 | 6 | 0.007 | 28 | NS | 28 | NS | 28 | 0.03 | 39 | NS |
| T2 | 36 | | 7 | | 25 | | 11 | | 46 | |
| T3 and T4 | 48 | | 24 | | 33 | | 46 | | 50 | |
| Lymphatic invasion[b] | | | | | | | | | | |
| Positive | 44 | NS | 19 | NS | 44 | NS | 33 | NS | 58 | 0.04 |
| Negative | 29 | | 20 | | 29 | | 31 | | 37 | |
| Venous invasion[b] | | | | | | | | | | |
| Positive | 54 | 0.02 | 15 | NS | 42 | 0.02 | 42 | NS | 62 | NS |
| Negative | 29 | | 21 | | 20 | | 27 | | 41 | |
| Lymph node metastasis[b] | | | | | | | | | | |
| Positive | 34 | NS | 14 | NS | 37 | NS | 34 | NS | 60 | 0.046 |
| Negative | 37 | | 23 | | 25 | | 30 | | 39 | |
| Distant metastasis[b] | | | | | | | | | | |
| Positive | 45 | NS | 15 | NS | 20 | NS | 40 | NS | 35 | NS |
| Negative | 33 | | 21 | | 32 | | 29 | | 50 | |

NS, Not significant; [a]Mann-Whitney's U-test; [b]chi-squared test or Fisher's exact probability test significantly higher in cases positive (55%, 16/29) for TIMP-2 in the tumour cell cytoplasm than in negative cases (27%, 17/63; $P < 0.01$; $\chi$-squared test). The percentage of cases positive for MMP-2 in the tumour cell cytoplasm was significantly higher in cases positive for TIMP-2 in the tumour cell cytoplasm (41%, 12/29) than in negative cases (10%, 6/63; $P < 0.01$; $\chi$-squared test). The frequency of samples positive for all three (MT1-MMP, MMP-2 and TIMP-2) was 12% (11/92), and the frequency of samples negative for all three was 23% (21/92).

### DISCUSSION

MMP-2, which is a type IV collagen-degrading enzyme, is a very important factor in the infiltration and metastasis of several carcinomas. TIMPs are intrinsic inhibitors of MMPs and have been studied clinically as potential carcinostatic agents (Watson et al, 1995; 1996; An et al, 1997). Theoretically, MMP-2 and TIMP-2 should be immunolocalized only in fibroblasts and monocytes at the sites that produce MMP-2 and TIMP-2 (Poulsom et al, 1992; Liabakk et al, 1996; Pyke et al, 1993; Ito et al, 1995). However, they have also been immunolocalized in the cytoplasm and cell membranes of cancer cells (Tomita and Iwata, 1996; Nomura et al, 1996; Höyhtyä et al. 1994). In our study, we found that MMP-2 and TIMP-2 were immunostained not only in the stroma of tumours but also in the cytoplasm of cancer cells, as well as there being elevated frequencies of expression of MT1-MMP, MMP-2, and TIMP-2 in the cytoplasm of cancer cells. One reason for this is that TIMP-2 and proMMP-2 might have been anchored to the cell membrane by MT1-MMP (Sato et al, 1996; Imai et al, 1996).

Expression of MMP-2 was not strongly correlated with factors related to infiltration apart from vascular invasion in our study. One explanation of our results is that the immunostaining with the MMP-2-specific antibody used in this study did not reflect the activity of MMP-2 since the antibody recognized both MMP-2 and proMMP-2 (Fujimoto et al, 1993). Furthermore, Liabakk et al (1996) reported that less-advanced tumours at Dukes' stage A have higher levels of active MMP-2 than do more invasive tumours at Dukes' stage B. These results raise the possibility that MMP-2 might be necessary while the tumour is in the process of penetrating the bowel wall in tumours at Dukes' stage A but might be less important when tumours have spread beyond muscle into the surrounding adipose tissue, as at stage B. Consequently, the immunohistochemical detection of MMP-2 appears not to be an appropriate indicator of infiltration and metastasis in a clinical setting.

The expression of TIMP-2 has been reported to be closely correlated with the progression of human colorectal cancer, a proposal that conflicts with the original function of TIMP-2 as an inhibitor of MMPs (Murashige et al, 1996; Tomita and Iwata, 1996). In our study, expression of TIMP-2 in the tumour cell cytoplasm was correlated with depth of invasion and expression of TIMP-2 in the stroma was correlated with lymphatic invasion and lymph node metastasis. These results indicate that TIMP-2 is a reliable indicator of the progression of human colorectal carcinoma. These results are in conflict with the original proposed functions of inhibitors of MMPs for the following reasons. TIMPs counteract the proteolytic functions of MMPs in a stoichiometric manner, at a ratio of 1:1 (Stetler-Stevenson et al, 1993*b*). However, the presence of excess TIMPs, as compared to MMPs, in tumour tissue might indicate that an abnormal ratio in tumour tissue was associated with growth and metastasis, and the binding and biological actions of MMPs and TIMPs might be altered in the presence of excess TIMPs (Tomita and Iwata, 1996; Kossakowska et al, 1996). In addition, the TIMP-2-specific antibody used in our study also recognized the proMMP-2/TIMP-2 complex (Höyhtyä et al, 1994). TIMP-2 in this complex functions as an activator of proMMP-2. Accordingly, under difficult circumstances, such as when quantifying TIMP-2 and MMP-2, immunohistochemical detection of TIMP-2 might indicate that TIMP-2 is essential for the activation of proMMP-2.

Recently, MT1-MMP was identified in the cell membranes of transfected cells that expressed MT-MMP and the expression of

*© 2000 Cancer Research Campaign*

MT1-MMP induced the activation of the precursor to MMP-2, proMMP-2 (Sato et al, 1994). There were few previous reports of immunohistochemical detection of MT1-MMP in colorectal carcinoma. In tumour cells of invasive lung carcinomas, MT1-MMP was immunolocalized in the carcinoma cells but not in the parenchymal or stromal cells of the surrounding normal tissue (Sato et al, 1994). In gastric carcinoma, MT1-MMP was predominantly immunolocalized in the carcinoma cells, and some carcinoma cells showed immunostaining on the cell membrane (Nomura et al, 1995). We found that MT1-MMP was immunolocalized predominantly in colorectal carcinoma cells, while its levels were low or it was absent in normal tissue. Moreover, expression of MT1-MMP was correlated with vascular invasion in cases of gastric carcinoma (Nomura et al, 1995) and with lymph node metastases in cases of lung carcinoma (Tokuraku et al, 1995) in previous studies. The present study showed that expression of MT1-MMP was correlated with vascular invasion and the depth of invasion. These results suggest that the expression of MT1-MMP might reflect infiltration in colorectal carcinoma.

Thus, immunohistochemical detection of MT1-MMP and TIMP-2 provides appropriate indices of infiltration but expression is not correlated with distant metastases in colorectal carcinoma. Our results indicate that not only MMPs but also other factors are required for the development of distant metastases.

### REFERENCES

An Z, Wang X, Willmott N, Chander SK, Tickle S, Docherty AJ, Mountain A, Millican AT, Morphy R, Porter JR, Epemolu RO, Kubota T, Moossa AR and Hoffman RM (1997) Conversion of highly malignant colon cancer from an aggressive to a controlled disease by oral administration of a metalloproteinase inhibitor. *Clin Exp Metastasis* **15**: 184–195

Bramhall SR, Stamp GW, Dunn J, Lemoine NR and Neoptolemos JP (1996) Expression of collagenase (MMP2), stromelysin (MMP3) and tissue inhibitor of the metalloproteinases (TIMP1) in pancreatic and ampullary disease. *Br J Cancer* **73**: 972–978

Collier IE, Wilhelm SM, Eisen AZ, Marmer BL, Grant GA, Seltzer JL, Kronberger A, He CS, Bauer EA and Goldberg GI (1997) H-ras oncogene-transformed human bronchial epithelial cells (TBE-1) secrete a single metalloprotease capable of degrading basement membrane collagen. *J Biol Chem* **263**: 6579–6587

Davies B, Miles DW, Happerfield LC, Naylor MS, Bobrow LG, Rubens RD and Balkwill FR (1993) Activity of type IV collagenases in benign and malignant breast disease. *Br J Cancer* **67**: 1126–1131

Fujimoto N, Mouri N, Iwata K, Ohuchi E, Okada Y and Hayakawa T (1993) A one-step sandwich enzyme immunoassay for human matrix metalloproteinase 2 (72-kDa gelatinase/type IV collagenase) using monoclonal antibodies. *Clin Chim Acta* **221**: 91–103

Hayakawa T (1994) Tissue inhibitors of metalloproteinases and their cell growth-promoting activity. *Cell Struct Funct* **19**: 109–114

Höyhtyä M, Fridman R, Komarek D, Porter-Jordan K, Stetler-Stevenson WG, Liotta LA and Liang CM (1994) Immunohistochemical localization of matrix metalloproteinase 2 and its specific inhibitor TIMP-2 in neoplastic tissues with monoclonal antibodies. *Int J Cancer* **56**: 500–505

Imai K, Ohuchi E, Aoki T, Nomura H, Fujii Y, Sato H, Seiki M and Okada Y (1996) Membrane-type matrix metalloproteinase 1 is a gelatinolytic enzyme and is secreted in a complex with tissue inhibitor of metalloproteinases 2. *Cancer Res* **56**: 2707–2710

Ito A, Nakajima S, Sasaguri Y, Nagase H and Mori Y (1995) Co-culture of human breast adenocarcinoma MCF-7 cells and human dermal fibroblasts enhances the production of matrix metalloproteinases 1, 2 and 3 in fibroblasts. *Br J Cancer* **71**: 1039–1045

Kossakowska AE, Huchcroft SA, Urbanski SJ and Edwards DR (1996) Comparative analysis of the expression patterns of metalloproteinases and their inhibitors in breast neoplasia, sporadic colorectal neoplasia, pulmonary carcinomas and malignant non-Hodgkin's lymphomas in humans. *Br J Cancer* **73**: 1401–1408

Liabakk NB, Talbot I, Smith RA, Wilkinson K and Balkwill F (1996) Matrix metalloprotease 2 (MMP-2) and matrix metalloprotease 9 (MMP-9) type IV collagenases in colorectal cancer. *Cancer Res* **56**: 190–196

Murashige M, Miyahara M, Shiraishi N, Saito T, Kohno K and Kobayashi M (1996) Enhanced expression of tissue inhibitors of metalloproteinases in human colorectal tumors. *Jpn J Clin Oncol* **26**: 303–309

Naylor MS, Stamp GW, Davies BD and Balkwill FR (1994) Expression and activity of MMPs and their regulators in ovarian cancer. *Int J Cancer* **58**: 50–56

Nomura H, Sato H, Seiki M, Mai M and Okada Y (1995) Expression of membrane-type matrix metalloproteinase in human gastric carcinomas. *Cancer Res* **55**: 3263–3266

Nomura H, Fujimoto N, Seiki M, Mai M and Okada Y (1996) Enhanced production of matrix metalloproteinases and activation of matrix metalloproteinase 2 (gelatinase A) in human gastric carcinomas. *Int J Cancer* **69**: 9–16

Poulsom R, Pignatelli M, Stetler-Stevenson WG, Liotta LA, Wright PA, Jeffery RE, Longcroft JM, Rogers L and Stamp GW (1992) Stromal expression of 72 kDa type IV collagenase (MMP-2) and TIMP-2 mRNAs in colorectal neoplasia. *Am J Pathol* **141**: 389–396

Pyke C, Ralfkiaer E, Tryggvason K and Dan K (1993) Messenger RNA for two type IV collagenases is located in stromal cells in human colon cancer. *Am J Pathol* **142**: 359–365

Ring P, Johansson K, Höyhtyä M, Rubin K and Lindmark G (1997) Expression of tissue inhibitor of metalloproteinases TIMP-2 in human colorectal cancer – a predictor of tumour stage. *Br J Cancer* **76**: 805–811

Sato H, Takino T, Okada Y, Cao J, Shinagawa A, Yamamoto E and Seiki M (1994) A matrix metalloproteinase expressed on the surface of invasive tumour cells. *Nature* **370**: 61–65

Sato H, Takino T, Kinoshita T, Imai K, Okada Y, Stetler Stevenson WG and Seiki M (1996) Cell surface binding and activation of gelatinase A induced by expression of membrane-type-1-matrix metalloproteinase (MT1-MMP). *FEBS Lett* **385**: 238–240

Sier CF, Kubben FJ, Ganesh S, Heerding MM, Griffioen G, Hanemaaijer R, van Krieken JH, Lamers CB and Verspaget HW (1996) Tissue levels of matrix metalloproteinases MMP-2 and MMP-9 are related to the overall survival of patients with gastric carcinoma. *Br J Cancer* **74**: 413–417

Sobin LH and Wittekind Ch (eds) (1997) TNM Classification of Malignant Tumours, 5th edn. pp. 66–69. Wiley-Liss: New York

Stetler-Stevenson WG, Liotta LA and Kleiner DE Jr (1993*a*) Extracellular matrix 6: role of matrix metalloproteinases in tumor invasion and metastasis. *FASEB J* **7**: 1434–1441

Stetler-Stevenson WG, Aznavoorian S and Liotta LA (1993*b*) Tumor cell interactions with the extracellular matrix during invasion and metastasis. *Annu Rev Cell Biol* **9**: 541–573

Tokuraku M, Sato H, Murakami S, Okada Y, Watanabe Y and Seiki M (1995) Activation of the precursor of gelatinase A/72 kDa type IV collagenase/MMP-2 in lung carcinomas correlates with the expression of membrane-type matrix metalloproteinase (MT-MMP) and with lymph node metastasis. *Int J Cancer* **64**: 355–359

Tomita T and Iwata K (1996) Matrix metalloproteinases and tissue inhibitors of metalloproteinases in colonic adenomas-adenocarcinomas. *Dis Colon Rectum* **39**: 1255–1264

Visscher DW, Höyhtyä M, Ottosen SK, Liang CM, Sarkar FH, Crissman JD and Fridman R (1994) Enhanced expression of tissue inhibitor of metalloproteinase-2 (TIMP-2) in the stroma of breast carcinomas correlates with tumor recurrence. *Int J Cancer* **59**: 339–344

Watson SA, Morris TM, Robinson G, Crimmin MJ, Brown PD and Hardcastle JD (1995) Inhibition of organ invasion by the matrix metalloproteinase inhibitor batimastat (BB-94) in two human colon carcinoma metastasis models. *Cancer Res* **55**: 3629–3633

Watson SA, Morris TM, Parsons SL, Steele RJ and Brown PD (1996) Therapeutic effect of the matrix metalloproteinase inhibitor, batimastat, in a human colorectal cancer ascites model. *Br J Cancer* **74**: 1354–1358

*© 2000 Cancer Research Campaign*

## References

[PDF_00272] An Z., Wang X., Willmott N., Chander S. K., Tickle S., Docherty A. J., Mountain A., Millican A. T., Morphy R., Porter J. R. (1997). Conversion of highly malignant colon cancer from an aggressive to a controlled disease by oral administration of a metalloproteinase inhibitor.. Clin Exp Metastasis.

[PDF_00277] Bramhall S. R., Stamp G. W., Dunn J., Lemoine N. R., Neoptolemos J. P. (1996). Expression of collagenase (MMP2), stromelysin (MMP3) and tissue inhibitor of the metalloproteinases (TIMP1) in pancreatic and ampullary disease.. Br J Cancer.

[PDF_00281] Collier I. E., Wilhelm S. M., Eisen A. Z., Marmer B. L., Grant G. A., Seltzer J. L., Kronberger A., He C. S., Bauer E. A., Goldberg G. I. (1988). H-ras oncogene-transformed human bronchial epithelial cells (TBE-1) secrete a single metalloprotease capable of degrading basement membrane collagen.. J Biol Chem.

[PDF_00286] Davies B., Miles D. W., Happerfield L. C., Naylor M. S., Bobrow L. G., Rubens R. D., Balkwill F. R. (1993). Activity of type IV collagenases in benign and malignant breast disease.. Br J Cancer.

[PDF_00289] Fujimoto N., Mouri N., Iwata K., Ohuchi E., Okada Y., Hayakawa T. (1993). A one-step sandwich enzyme immunoassay for human matrix metalloproteinase 2 (72-kDa gelatinase/type IV collagenase) using monoclonal antibodies.. Clin Chim Acta.

[PDF_00293] Hayakawa T. (1994). Tissue inhibitors of metalloproteinases and their cell growth-promoting activity.. Cell Struct Funct.

[PDF_00295] Höyhtyä M., Fridman R., Komarek D., Porter-Jordan K., Stetler-Stevenson W. G., Liotta L. A., Liang C. M. (1994). Immunohistochemical localization of matrix metalloproteinase 2 and its specific inhibitor TIMP-2 in neoplastic tissues with monoclonal antibodies.. Int J Cancer.

[PDF_00299] Imai K., Ohuchi E., Aoki T., Nomura H., Fujii Y., Sato H., Seiki M., Okada Y. (1996). Membrane-type matrix metalloproteinase 1 is a gelatinolytic enzyme and is secreted in a complex with tissue inhibitor of metalloproteinases 2.. Cancer Res.

[PDF_00303] Ito A., Nakajima S., Sasaguri Y., Nagase H., Mori Y. (1995). Co-culture of human breast adenocarcinoma MCF-7 cells and human dermal fibroblasts enhances the production of matrix metalloproteinases 1, 2 and 3 in fibroblasts.. Br J Cancer.

[PDF_00307] Kossakowska A. E., Huchcroft S. A., Urbanski S. J., Edwards D. R. (1996). Comparative analysis of the expression patterns of metalloproteinases and their inhibitors in breast neoplasia, sporadic colorectal neoplasia, pulmonary carcinomas and malignant non-Hodgkin's lymphomas in humans.. Br J Cancer.

[PDF_00312] Liabakk N. B., Talbot I., Smith R. A., Wilkinson K., Balkwill F. (1996). Matrix metalloprotease 2 (MMP-2) and matrix metalloprotease 9 (MMP-9) type IV collagenases in colorectal cancer.. Cancer Res.

[PDF_00315] Murashige M., Miyahara M., Shiraishi N., Saito T., Kohno K., Kobayashi M. (1996). Enhanced expression of tissue inhibitors of metalloproteinases in human colorectal tumors.. Jpn J Clin Oncol.

[PDF_00318] Naylor M. S., Stamp G. W., Davies B. D., Balkwill F. R. (1994). Expression and activity of MMPS and their regulators in ovarian cancer.. Int J Cancer.

[PDF_00323] Nomura H., Fujimoto N., Seiki M., Mai M., Okada Y. (1996). Enhanced production of matrix metalloproteinases and activation of matrix metalloproteinase 2 (gelatinase A) in human gastric carcinomas.. Int J Cancer.

[PDF_00320] Nomura H., Sato H., Seiki M., Mai M., Okada Y. (1995). Expression of membrane-type matrix metalloproteinase in human gastric carcinomas.. Cancer Res.

[PDF_00326] Poulsom R., Pignatelli M., Stetler-Stevenson W. G., Liotta L. A., Wright P. A., Jeffery R. E., Longcroft J. M., Rogers L., Stamp G. W. (1992). Stromal expression of 72 kda type IV collagenase (MMP-2) and TIMP-2 mRNAs in colorectal neoplasia.. Am J Pathol.

[PDF_00330] Pyke C., Ralfkiaer E., Tryggvason K., Danø K. (1993). Messenger RNA for two type IV collagenases is located in stromal cells in human colon cancer.. Am J Pathol.

[PDF_00333] Ring P., Johansson K., Höyhtyä M., Rubin K., Lindmark G. (1997). Expression of tissue inhibitor of metalloproteinases TIMP-2 in human colorectal cancer--a predictor of tumour stage.. Br J Cancer.

[PDF_00339] Sato H., Takino T., Kinoshita T., Imai K., Okada Y., Stetler Stevenson W. G., Seiki M. (1996). Cell surface binding and activation of gelatinase A induced by expression of membrane-type-1-matrix metalloproteinase (MT1-MMP).. FEBS Lett.

[PDF_00336] Sato H., Takino T., Okada Y., Cao J., Shinagawa A., Yamamoto E., Seiki M. (1994). A matrix metalloproteinase expressed on the surface of invasive tumour cells.. Nature.

[PDF_00343] Sier C. F., Kubben F. J., Ganesh S., Heerding M. M., Griffioen G., Hanemaaijer R., van Krieken J. H., Lamers C. B., Verspaget H. W. (1996). Tissue levels of matrix metalloproteinases MMP-2 and MMP-9 are related to the overall survival of patients with gastric carcinoma.. Br J Cancer.

[PDF_00352] Stetler-Stevenson W. G., Aznavoorian S., Liotta L. A. (1993). Tumor cell interactions with the extracellular matrix during invasion and metastasis.. Annu Rev Cell Biol.

[PDF_00349] Stetler-Stevenson W. G., Liotta L. A., Kleiner D. E. (1993). Extracellular matrix 6: role of matrix metalloproteinases in tumor invasion and metastasis.. FASEB J.

[PDF_00355] Tokuraku M., Sato H., Murakami S., Okada Y., Watanabe Y., Seiki M. (1995). Activation of the precursor of gelatinase A/72 kDa type IV collagenase/MMP-2 in lung carcinomas correlates with the expression of membrane-type matrix metalloproteinase (MT-MMP) and with lymph node metastasis.. Int J Cancer.

[PDF_00360] Tomita T., Iwata K. (1996). Matrix metalloproteinases and tissue inhibitors of metalloproteinases in colonic adenomas-adenocarcinomas.. Dis Colon Rectum.

[PDF_00363] Visscher D. W., Höyhtyä M., Ottosen S. K., Liang C. M., Sarkar F. H., Crissman J. D., Fridman R. (1994). Enhanced expression of tissue inhibitor of metalloproteinase-2 (TIMP-2) in the stroma of breast carcinomas correlates with tumor recurrence.. Int J Cancer.

[PDF_00371] Watson S. A., Morris T. M., Parsons S. L., Steele R. J., Brown P. D. (1996). Therapeutic effect of the matrix metalloproteinase inhibitor, batimastat, in a human colorectal cancer ascites model.. Br J Cancer.

[PDF_00367] Watson S. A., Morris T. M., Robinson G., Crimmin M. J., Brown P. D., Hardcastle J. D. (1995). Inhibition of organ invasion by the matrix metalloproteinase inhibitor batimastat (BB-94) in two human colon carcinoma metastasis models.. Cancer Res.

